# Fluctuations of dissociation and inner tension during inpatient dialectical behavior therapy: associations with self-injury and suicidal ideation

**DOI:** 10.1186/s40479-026-00339-1

**Published:** 2026-03-06

**Authors:** Ana Macchia, Dimitri Löchner, Christian Montag, Philip Santangelo, Michelle Voit, Simon Sanwald, Birgit Abler

**Affiliations:** 1 Department of Psychiatry/Psychotherapy III, Ulm University Clinic, Leimgrubenweg 12-24, 89075 Ulm, Germany; 2 Institute of Psychology, University of Tartu, Tartu, Estonia; 3 Centre for Cognitive and Brain Sciences, Institute of Collaborative Innovation, University of Macau, Macau SAR, China; 4 Department of Computer and Information Science, Faculty of Science and Technology, University of Macau, Macau SAR, China; 5 Department of Psychology, Faculty of Social Sciences, University of Macau, Macau SAR, China; 6 Department of Behavioural and Cognitive Sciences, Luxembourg University, Luxembourg, Luxembourg

**Keywords:** Ecological momentary assessment (EMA), Dialectic Behavior Therapy (DBT), Borderline personality disorder (BPD), Inner tension and affective instability, State dissociation

## Abstract

**Background:**

In Dialectical Behavior Therapy (DBT), monitoring inner tension and state dissociation is considered helpful for understanding a patient’s affective and behavioral responses, especially regarding self-harming behaviors. This study aims to investigate how potentially aversive internal states including dissociation, inner tension and fluctuations of tension, i.e. affective instability, during the initial phase of inpatient DBT relate to self-injury, suicidal ideation, and symptom reduction.

**Methods:**

41 patients with personality disorder (borderline or combined), undergoing an 8-week inpatient DBT program used a smartphone application for ecological momentary assessment of inner tension and state dissociation. We assessed the Borderline Symptom List (BSL-23) upon admission and discharge, self-injury during the inpatient stay, and the intensity of suicidal ideation per day (diary card ratings) retrospectively from digital patient files. We employed linear mixed models to analyze the trajectories of inner tension, affective instability (i.e. squared difference between consecutive inner tension ratings), and state dissociation over the initial 3 weeks of therapy and variability across hours, as well as daily associations with the intensity of suicidal ideation. We used logistic regression to examine if aversive internal states are associated with the occurrence of self-injury during DBT.

**Results:**

We found a slight reduction in state dissociation, affective instability, and high levels of inner tension during the initial 3 weeks of therapy. Analyses of intraday data showed a slight increase in state dissociation from morning to midday and self-injury during DBT was associated with slightly higher mean dissociation levels. On days with greater intensity of suicidal ideation, elevated inner tension and state dissociation levels were found while affective instability was not related to self-injury or suicidal ideation. Changes in BSL-23 over therapy were not related to aversive internal states during the initial therapy phase.

**Conclusion:**

Aversive internal states decreased early in inpatient DBT, with midday emerging as a critical time for dissociation management. Daily increases in state dissociation and inner tension might serve as warning signs for suicidality and our data confirm the utility of their monitoring in self-harm prevention. When state dissociation is therapeutically addressed during DBT as in the program investigated, it does not seem to hamper improvement in borderline symptoms.

**Trial registration:**

Retrospectively registered - https://osf.io/dfq9y/?view_only=4c19b891bb6448009b22f60b2552bd73.

**Supplementary Information:**

The online version contains supplementary material available at 10.1186/s40479-026-00339-1.

## Introduction

In behavioral therapy, it is assumed that internal states—such as emotions, bodily sensations, and thoughts—are closely linked to observable behaviors. Understanding and addressing internal states throughout treatment are thought to facilitate behavioral change -and vice versa. In individuals with borderline personality disorder (BPD), internal states often manifest as undifferentiated negative affect, which describes the immediate, momentary experience of negative emotions as diffuse and poorly differentiated, reflecting low emotional granularity or emotional complexity. Instead of distinguishing between discrete emotions (e.g. anger, sadness), individuals experience a generalized sense of unpleasant feelings [1]. Because discrete emotions provide information about situational demands and appropriate coping responses, reduced emotional differentiation may limit the ability to regulate emotions effectively. Accordingly, undifferentiated negative affect is conceptualized as a marker of emotion dysregulation or diminished regulatory capacity [1].

In BPD, such diffuse negative emotional states are frequently accompanied by elevated levels of aversive inner tension, a subjectively experienced state of heightened emotional arousal characterized by feelings of pressure, agitation, or being overwhelmed. While undifferentiated negative affect captures the qualitative indistinctness of emotional experience, aversive inner tension reflects its subjectively experienced intensity dimension. Feelings of aversive inner tension may hinder adaptive behavior and promote impulsive actions in BPD [2]. Thus, dysfunctional emotion regulation leading to heightened and prolonged states of inner tension, has been linked to self-harming behaviors [3]. Monitoring levels of inner tension may help patients learn to differentiate emotions and recognize triggers for dysfunctional behavior, and it is especially encouraged during the initial phase of psychotherapy of BPD [4, 5]. While increases of inner tension at medium levels can be functional when coping with situations requiring increased attention and arousal, excessive inner tension (associated with hyperarousal, heightened emotionality and loss of control) [6] is seen as a typical aspect of BPD psychopathology.

Hereby, not only increases of inner tension but particularly its rapid fluctuations, often driven by polarized emotional experiences of “feeling all good or all bad” [7] have been identified as characteristic for BPD [8]. High affective instability, defined as extreme, frequent, and unpredictable changes of mood (i.e., a prolonged emotional state with low situational specificity) over time [8] significantly impacts other BPD symptoms [8, 9] and might also contribute to suicidality [10]. However, while baseline affective instability (DSM Criteria) revealed to be an important predictor of self-injury in the general population [11], data on how momentary affective instability relates to various self-harming behaviors in BPD are more scarce. Links et al., [12] found that negative mood intensity, but not affective instability significantly related to the intensity of self-reported suicidal ideation and the past number of suicide attempts. These findings may suggest that rather states of high inner tension than rapid shifts in affectivity might be associated with suicidality and self-injury. Zaki et al., [13] showed that BPD patients high in negative emotional differentiation (i.e., the ability to distinguish between similar emotions, such as sadness and anger, rather than describing feelings in broad terms) are at a lower risk for self-injury compared to those with low negative emotion differentiation, also suggesting that the experience of high inner tension might relate to self-injury.

Self-injury may serve multiple functions, occurring either in response to aversive internal states or to achieve social goals — aiming to reduce negative emotions or to generate positive sensations [14]. In the context of the “Nonparadoxical Theory of the Functions of Self-Harm” [15] dissociation going along with disconnect between emotion, body, and self may be another trigger for self-harming behavior with the latter counteracting the disconnection. Clinically relevant levels of dissociation represent a specific symptom occurring in only a subset of BPD patients and are directly targeted in DBT through behavioural analyses and tailored skills [4, 16]. According to the theory, self-harm helps to restore emotional clarity, bodily presence, and a sense of reality, whether individuals feel overwhelmed by emotions or numb, thereby interrupting dissociative states and creating distance from intense inner tension to regain control and reconnect the self. Concordantly, dissociative symptoms emerged as a significant long-term risk factor for self-injury [17] and higher lifetime frequency of self-injury and self-injury in the past year before DBT is significantly associated with heightened dissociation [18, 19]. This suggests that dissociation may play a central role in the development and maintenance of self-injurious behavior. However, the nature of this association remains unclear. Some findings suggest that the relationship weakens after controlling for overall symptom severity, indicating that dissociation may partly reflect broader psychopathology [20]. At the same time, experimental and clinical studies point to potential mechanisms through which dissociation may facilitate self-injury, including reduced pain perception and a reduction in aversive inner tension following tissue damage [3, 21, 22]. In other patients, self-injury might be perceived as a strategy to regulate dissociative states [23].

A less intensively researched area is the relationship of inner tension and state dissociation with suicidal ideation. A meta-analysis found a moderate positive relationship between dissociation and suicidal ideation (*r* = .32), highlighting a significant link that has been overlooked in suicide research [24]. In a network analysis of a representative sample of 3,500 young adults from the Scottish Wellbeing Study, internal entrapment, the feeling of being trapped by one’s own thoughts and emotions, and perceived burdensomeness emerged as the factors most closely linked to suicidal ideation. Suicidal ideation may reflect a state of intense psychological strain that might be conceptually similar to feelings of entrapment [25]. These results suggest a potential association between states of inner tension and suicidal ideation in patients with BPD.

Moreover, previous studies found diverging results regarding a potentially impairing effect of trait dissociation on therapeutic success. Trait dissociation, understood as a stable tendency to experience dissociative symptoms across contexts, can be distinguished from state dissociation, which refers to transient, context-dependent dissociative experiences [26]. Contradictory results might relate to different outcome measures (symptoms of posttraumatic stress disorder/PTSD or BPD) used, diverging definitions of dissociation, different settings (RCT vs. naturalistic) and different psychometric instruments to assess dissociation [19, 27, 28]. In contrast, the role of state dissociation in predicting therapeutic outcomes has received comparatively little attention in research. A study by Kleindienst et al., [29] found that patients with low state dissociation during treatment had a higher chance to show substantial improvement in PTSD symptoms. This relation consistently emerged across subgroups of PTSD patients with and without BPD. In another study, Kleindienst et al., [30] found that baseline state dissociation assessed with the dissociation tension scale was a negative predictor for outpatient treatment efficacy, operationalized as a change in the Clinician-Administered PTSD Scale, in individuals suffering from complex PTSD treated with an adaptation of DBT focussing on trauma (DBT-PTSD). However, it remains unclear whether state dissociative symptoms also have an impact on changes in borderline symptomatology (BSL-23) in patients undergoing standard DBT.

### Study aim and hypotheses

Despite the common understanding of internal states (i.e., inner tension, affective instability, state dissociation) as potential triggers or precursors of problematic behavior like suicidal ideation and self-injury, their momentary connections in real life remain poorly understood. Clarifying this link could be especially useful in inpatient or intensive outpatient settings, where therapeutic interventions might be tailored to address these fluctuating internal states more effectively. Furthermore, little is known about how fluctuations of inner states unfold in real time over the course of the day and throughout therapy. Understanding these patterns may help to better identify when interventions are most needed and whether the intensity relates to therapeutic success since little is known through which mechanisms DBT exerts its effects on a psychological level.

This naturalistic study primarily aims to shed light on fluctuations of internal states during the first three weeks of an 8 to 10-week inpatient DBT program, both across and within days, using Ecological Momentary Assessment (EMA). We focused on the first three weeks of therapy because compliance with self-monitoring was sufficiently high during this period, and self-monitoring is particularly encouraged in the initial phase of DBT. We were interested in whether and if so, to what extent, inner tension, affective instability, and state dissociation in the initial phase of inpatient therapy are associated with self-injury and suicidal ideation, as well as symptom reduction during DBT.

## Methods

### Sample and procedure

The initial study sample consisted of 99 patients who underwent an 8 to 10-week certified (Dachverband DBT e.V.) inpatient DBT program at the Clinic for Psychiatry/Psychotherapy III, Ulm University, Germany (data collection between April 2022 and June 2023). All patients participating in the DBT program who had an Android operating system were eligible for study participation (this is due to problems in providing some of the app’s features to the more restricted iOS environment). Diagnosis of BPD were assessed by a senior physician (B.A.) using the International Personality Disorder Examination of the ICD-10 (IPDE) which is a semi-structured interview that can be used to diagnose a personality disorder [31]. 59 patients (60% of all eligible patients) opted for digital monitoring with the *Insights* app (www.insightsapp.org, [32, 33]) instead of using the paper-and-pencil version to track inner tension and state dissociation throughout the therapy.

The Ethics Committee of the University of Ulm, Germany (EA 301/21) approved the study protocol. Rights of the subjects are protected by written informed consent to participate and all provided written informed consent in accordance with the Declaration of Helsinki and ethical guidelines. Of the eligible patients, 55 had the main diagnosis of a personality disorder. 4 patients that opted for using the app as part of the treatment program had other main diagnoses (PTSD, major depression, substance abuse, mild mental retardation). The first three weeks of therapy, where patients were particularly encouraged to monitor internal states as part of the therapy program, were analyzed, though patients could use the app for the whole inpatient stay. In the analysis presented here, we only included patients with monitoring days with a compliance rate of at least 70% (5 ratings/day: compliant treatment day) during the first three weeks of the therapy. Compliance rates in EMA studies typically range between approximately 70% and 80%, with values around 80% often considered a gold standard in intensive longitudinal research [34, 35]. We therefore defined a compliance threshold of 70% to ensure reliable estimation of within-person processes while retaining sufficient data for analysis and minimizing the risk of systematic bias due to sparse individual time series. To assess reliability of our data, control analyses were also conducted on the full dataset without the 70% compliance threshold, as no differences were observed, the more restrictive approach is reported here. The final sample consisted of 41 patients with personality disorder (4 men, 35 women, 2 transgender; age 18 to 41 years; *M* = 26.39 years, *SD* = 6.58 years). Of these patients, 37 were diagnosed with emotionally unstable personality disorder, borderline type, 2 with emotionally unstable personality disorder of the impulsive type, and 2 with a combined personality disorder predominantly featuring borderline traits (see Table S1). 12 patients (29.3%) had a higher education entrance qualification (equivalent to the German Abitur or Fachhochschulreife), 29 patients had a secondary school certificate (70.7%, i.e., 9-10 years of schooling). The majority of patients were not in a romantic relationship (*n* = 13, 35.1%) and were unmarried (*n* = 7, 18.9%). Two patients discontinued therapy before completing 21 monitoring days after 6 and 20 days respectively. In the 41 included patients, we could analyze on average 11.2 compliant treatment days (*SD* = 6.95, Range: 1–21), with 3547 total valid ratings and an average compliance rate (the number of recordings per day divided by 7, which is the number of daily reminders) of 95.55% (*SD* = 15.58). There was no financial compensation for study participation.

### Instruments

#### Ecological momentary assessment

Patients answered questions about their intensity of state dissociation and inner tension with their own smartphones using the *Insights* app (www.insightsapp.org, [32, 33]). Patients repeatedly rated their momentary intensity of inner tension and state dissociation throughout the entire treatment period of the DBT program on a Likert scale ranging from 0 (no tension/no dissociative phenomena) to 10 (maximal perceivable inner tension; absolute loss of control/maximal dissociation) with their own mobile devices. State dissociative phenomena were explained to the patients with a clinical definition containing the following information (but not the exact wording, as the information was conveyed in German and varied slightly between staff): “In case of dissociation, the brain ‘gets out of the way’. It is a survival function. One does not perceive the self/environment properly anymore” and exemplary symptoms listed in the Dissociative Experience Scale (DES-II, see below), which all patients had filled upon enrollment. Data entry was possible at any time of the day (but not retrospectively) and patients were instructed that they could answer the questions once every hour. The app reminded patients to fill out the questionnaires every two hours from 9.00 am to 9.00 pm resulting in seven daily reminders. 

#### Questionnaires

To assess BPD symptoms at admission (baseline, T0) and the end of treatment (T1), we included the short version of the German Borderline Symptom List (BSL-23; [36]), which is a self-rating questionnaire with 23 items. Further information on additional questionnaires used in this study is provided elsewhere and reliability analyses demonstrated high internal consistency in the current sample, including excellent internal consistency for the BSL-23 [37].

#### Self-injury, suicidal thoughts before DBT

Psychologists holding a master’s degree or trained students of medicine in a higher semester (> 6th semester) conducted a clinical interview with study patients at T0 to assess the following self-harm variables: Number of self-injuries during the last year, lifetime self-injuries, frequency of suicidal ideation during the past 6 months (average number of days with suicidal ideation per week).

#### Self-injury, suicidal thoughts during DBT

We assessed actual self-injury and the intensity of suicidal ideation per day during the DBT inpatient stay and entries from patients’ diary cards [4, 38] retrospectively from the staff’s documentation in digital patient files. Self-injury refers to any act in which a person intentionally causes physical harm or injury to themselves, without suicidal intent. We collected the date and frequency of self-injury during inpatient therapy and self-injury methods. Diary card data was collected as a regular part of the DBT program. It included daily assessments of the intensity of suicidal ideation on a Likert scale from 0 (no/not at all) to 5 (very high/very much). The nursing staff documented the assessments in the diary card daily from Monday to Friday, based on short therapeutic conversations with the patients taking place in the form of individual meetings between 5 p.m. and 6 p.m. each day.

### Statistical analyses

Data processing and statistical analyses were performed using R Version 4.3.1 [39]. Bayesian data analysis was conducted with JASP Version 0.19.3 [40]. We used heatplots to illustrate our longitudinal data per participant. Aggregated descriptive data were calculated in two steps. First, daily mean levels of inner tension and state dissociation were computed for each participant based on all available ratings within a day. Second, during the Initial Therapy Phase (ITP), individual state levels were obtained by averaging these daily means across the first three weeks of therapy. Only days with a compliance rate of at least 70% (i.e., 5 ratings per day) were included. This threshold was chosen to reduce the impact of missing data and ensure that daily averages were based on sufficiently reliable information. 

 To determine the level of affective instability, the squared difference of neighboring individual ratings that were not more than 1–2 h apart was calculated, representing an established instability index [8, 41]. 70.34% (*N* = 2495) of all ratings had a direct neighbor as defined here and could be included in the calculation of affective instability.

**Course of inner tension and state dissociation during therapy (longitudinal analyses)**: We employed linear mixed models (LMMs) to analyze the level of aversive internal states (i.e., inner tension, affective instability, and dissociation) depending on measurement day (i.e., first day of monitoring, usually day 2 or 3 after admission, continuous variable, 1 to 21), the number of app ratings (continuous variable), and weekend days which include Saturday and Sunday (dummy coded: 1 = weekend, 0 = weekdays). The models only included hours from 9 a.m. to 9 p.m. and account for variations in each variable level across patients by including random intercepts. We further included the interaction of measurement day x number of daily app ratings if it improved the model fit. Additionally, we explored a model based on the assumption that therapy might decrease high (Likert scale 6, 7) and particularly very high (Likert scale 8, 9, 10, indicating loss of control) values of inner tension which can be assumed as dysfunctional in most everyday life situations in favor to the relative number of ratings in the low to medium (0 to 4) range. The cutoff of 5 reflects both the midpoint of the 0–10 scale and the approximate median of inner tension in the present sample [37], providing a pragmatic distinction between lower and higher inner tension states. To describe fluctuations of internal states across different daytimes we calculated participant-centered means (i.e., aggregation per hour and participant as a first step and aggregation per hour as a second step) which accounts for the fact that some patients have more ratings per hour than others do. To analyze individual raw data, we employed LMMs with reminded hours (factor with 7 levels, 9 a.m. as reference) and measurement day (continuous variable) as fixed effects and a random intercept for patients. LMMs were analyzed via the restricted maximum likelihood method using the ‘lme4’ R-package [42]. Continuous variables of regression models were z-transformed (*M* = 0, *SD* = 1). *P*-values for regression coefficients were calculated using the ‘lmerTest’ package [43], which applies Satterthwaite’s approximation to estimate denominator degrees of freedom. We applied 1,000 resamples of bootstrapping on beta estimates to proof the robustness [44] of our linear mixed models and checked each model for testing assumptions including outliers with cook’s distance.

**Self-injury, suicidal ideation, inner tension, and state dissociation before DBT (cross-sectional analyses):** Associations of self-harm before DBT (i.e., interview assessed self-injury during the last year/lifetime, the frequency of suicidal ideation (average number of days with suicidal ideation per week), and participant-centered ITP levels of inner tension, affective instability, and state dissociation were explored by Spearman’s correlations to account for non-normality of the data. We applied 1,000 resamples of bootstrapping to estimate the confidence intervals, CI. We identified extreme outliers (defined as values falling below the first quartile minus 1.5 times the interquartile range or above the third quartile plus 1.5 times the interquartile range) in self-injuries one year before DBT (*n* = 5), lifetime self-injuries (*n* = 7), and affective instability (*n* = 1) and replaced them with trimmed mean values.

**Self-injury**,** inner tension and state dissociation during DBT (cross-sectional analyses)**: Cross-sectional analyses were chosen to investigate the influence of internal states on self-injury during therapy as assessed from patients’ files, as there was no simultaneous collection of internal states (collected by app) and self-injury (during therapy, patient’s files) variables. We used logistic regression and calculated odds ratios to analyze if participant-centered ITP mean internal state levels (continuous variables) relate to self-injury during therapy (dummy coded: 1 = occurrence of self-injury, 0 = no self-injury).

**Suicidal ideation, inner tension and state dissociation during DBT (longitudinal analyses):** Our longitudinal analyses investigated the effect of participant-centered daily internal states on daily ratings (scale from 0 to 5) of the intensity of suicidal ideation in the diary card using LMMs. Due to the structure of the diary card data (ratings from Monday to Friday), the models do not include weekend days. The first measurement day was defined as the first Monday of the inpatient stay on which tracking began. The effect on suicidal ideation assessed with the diary card was estimated separately for inner tension, state dissociation, and affective instability including measurement days (continuous variable). The models included random intercepts and slopes per participant.

**Inner tension, state dissociation and symptom reduction during therapy:** We employed simple linear regressions to investigate if the change in borderline-specific Symptoms from pre- to post therapy (Δ BSL = BSL T1 – BSL T0) relates to the level of ITP internal states, accounting for the baseline symptom severity (BSL T0). Missing data included *n* = 6 patients for the baseline assessment (T0) and *n* = 8 patients for the assessment pre-therapy (T1). To address missing data, multiple imputation was performed for post-therapy values using the mice (multivariate imputation by chained equations; [45]) function and the “predictive mean matching” (pmm) method was used to generate realistic values and avoid bias from linear assumptions.

Due to the small sample size, we additionally report Bayes factors for the correlations in the cross-sectional analyses to assess the evidence for the alternative hypothesis (BF₁₀). Missing data were handled using pairwise deletion, consistent with the approach used in the spearman correlation analyses. For the logistic and linear regressions, we report the Bayes factor comparing the alternative model to the null model (BF_M_). We interpreted the Bayes factors according to established thresholds, with values between 1 and 3 considered anecdotal evidence, values between 3 and 10 as moderate evidence, and values above 10 as strong evidence in favor of an effect [46].

## Results

### Course of inner tension and state dissociation during therapy (longitudinal analyses)

A LMM revealed no significant change of inner tension throughout the ITP (21 days), the number of app ratings did not relate to the level of inner tension, but inner tension levels were slightly lower on weekends (Table 1). Model-estimated inner tension showed predicted mean values of $\hat{M}$ = 4.12 (*SE* = 0.23, 95% CI [3.67, 4.56]) at measurement day 1 and $\hat{M}$ = 4.11 (*SE* = 0.24, 95% CI [3.65, 4.57]) at measurement day 21, corresponding to a negligible mean change of Δ = −0.01 points on the 0–10 scale. Interestingly, high and very high levels of inner tension (> 5) significantly decreased, β = -0.12 (95%CI: -0.18 to -0.05), *p* < .001) whereas average ratings in the low to medium range (< 5) increased significantly during the ITP, β = 0.15 (95%CI: 0.11 to 0.19), *p* < .001. We found a slight but significant reduction of state dissociation throughout the ITP and the number of app ratings was positively associated with state dissociation (more ratings in patients with higher values), while weekend days did not influence the level of state dissociation (Table 1). Model-estimated dissociation decreased from $\hat{M}$ = 1.77 (*SE* = 0.28, 95% CI [1.23, 2.32]) at measurement day 1 to $\hat{M}$ = 1.55 (*SE* = 0.28, 95% CI [1.00, 2.10]) at measurement day 21, corresponding to an average reduction of Δ = −0.22 points on the 0-10 scale. The model including the interaction of the number of app ratings and measurement days significantly improved the model fit, *F*(1,3124) = 16.43, *p*<.001 and revealed that those study patients with more app recordings on later treatment days had significantly higher levels of state dissociation (Table 1). We found a reduction of affective instability (squared successive difference between consecutive ratings 1–2 h apart of inner tension) throughout the ITP. Model-estimated values decreased from $\hat{M}$ = 3.31 (*SE* = 0.28, 95% CI [2.76, 3.86]) at measurement day 1 to $\hat{M}$ = 1.75 (SE = 0.35, 95% CI [1.05, 2.45]) at measurement day 21, corresponding to a mean reduction of Δ = −1.56 points (scale 0–64 square sum values). The number of app recordings and weekend days did not significantly influence the level of affective instability (all *p*’s > 0.58) (Table 1, see Table S2-S4 for details regarding LMMs and Figure S1-S2 for heatplots).

**Table 1:** Linear mixed model results (fixed effects) for inner tension, state dissociation, and affective instability as a function of the number of app ratings, measurement day, and weekend days

Predictors	b	β	95% CI	standardized CI	*p*
**Inner tension**					
(Intercept)	4.05	-0.11	3.58–4.53	-0.34–0.12	**< 0.001**
N	0.12	0.04	-0.02–0.27	-0.01–0.08	0.088
measurement day	-0.00	-0.00	-0.06–0.06	-0.03–0.03	0.922
weekend [1]	-0.16	-0.08	-0.28 – -0.03	-0.14 – -0.02	**0.014**
**State dissociation**					
(Intercept)	1.40	0.02	0.84–1.97	-0.24–0.28	**< 0.001**
N	0.23	0.05	0.10–0.36	0.02–0.09	**< 0.001**
measurement day	-0.25	-0.03	-0.35 – -0.14	-0.05 – -0.01	**< 0.001**
weekend [1]	-0.01	-0.00	-0.10–0.09	-0.05–0.04	0.894
N × measurement day	0.16	0.04	0.07–0.25	0.02–0.07	**0.001**
**Affective instability**					
(Intercept)	2.66	0.02	1.95–3.37	-0.08–0.11	**< 0.001**
N	-0.05	-0.01	-0.54–0.43	-0.07–0.05	0.826
measurement day	-0.47	-0.09	-0.70 – -0.25	-0.13 – -0.05	**< 0.001**
weekend [1]	-0.13	-0.03	-0.60–0.34	-0.12–0.07	0.582

*Note. b* = unstandardized regression coefficients; β = standardized regression coefficients; 95% CI = 95% confidence interval for unstandardized estimates; standardized CI = 95% confidence interval for standardized coefficients; *p* = *p* value. *N* = total number of ratings. Measurement day = DBT treatment day (continuous variable; 0-21). Weekend (1 = weekend, 0 = weekday). Continuous variables are z-transformed (mean = 0, sd = 1)

**Fig. 1:**
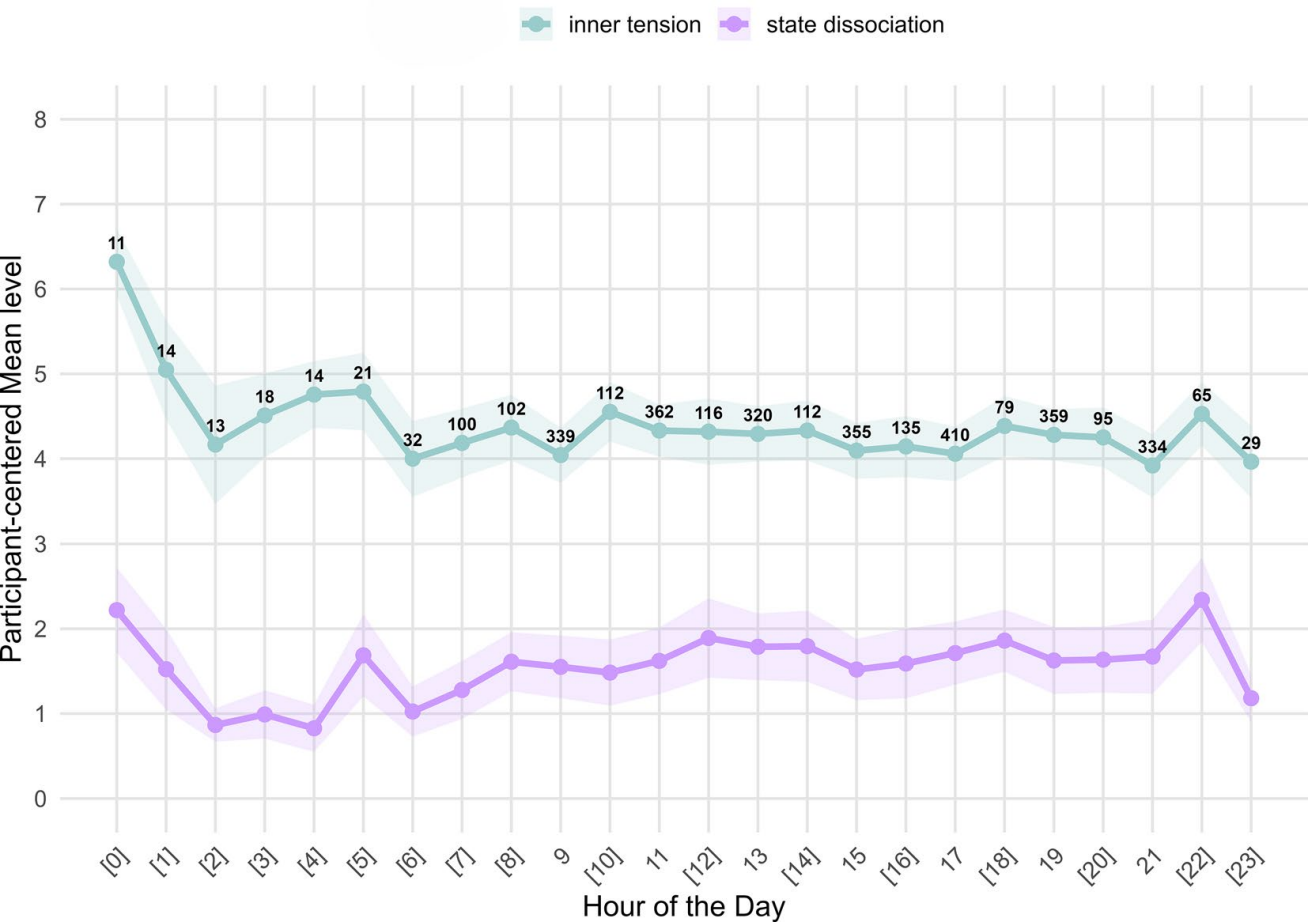
Participant-centered means and confidence intervals (first 21 monitored treatment days, ITP) for the levels of inner tension (blue line) and state dissociation (purple line) across different times of the day including the number of available data points (ratings) per hour for both inner tension and state dissociation. Hours in square brackets indicate hours for which the app did not send a reminder; hours without brackets indicate hours with a reminder. Numbers presented state how many rating where available at the respective time of the day (number for inner tension and state dissociation being the same)

Participant-centered mean values revealed the highest levels of dissociation at 10 p.m. and midnight (*M* = 2.34, *SD* = 2.43, *N*(ratings) = 65; *M* = 2.22, *SD* = 2.45, *N*(ratings) = 11), whereas lowest values were found from 2 to 4 a.m. and at 6 a.m. (see Fig. 1). Highest participant-centered mean inner tension levels were also found at midnight and 1 a.m. (*M* = 6.32, *SD* = 1.92, *N*(ratings) = 11; *M* = 5.05, *SD* = 2.85, *N*(ratings) = 14). However, findings during nighttime without prompts between 11 p.m. and 6 a.m. have to be treated with extreme caution due to a very low number of data collected at these times.

By analyzing the individual raw data per participant across prompted time points of the day between 9 a.m. and 9 p.m. (i.e., 9 a.m., 11 a.m., 1 p.m., etc.) for state dissociation, we found a significant increase from 9 a.m. to 11 a.m. (β = 0.08, 95% CI [0.01, 0.16], *b* = 0.18, bias corrected accelerated confidence interval, BCa CI 95% [0.03, 0.35], *p* = .018) and from 9 a.m. to 1 p.m. (β = 0.13, 95% CI [0.05, 0.21], *b* = 0.28, BCa CI 95% [0.12, 0.44], *p* < .001). There were no significant changes of inner tension and affective instability throughout the day when analyzing prompted ratings (see Table S5-S7).

### Self-injury, suicidal ideation, inner tension and state dissociation before DBT (cross-sectional analyses)

Descriptive statistics of self-harm, borderline symptoms before admission, and mean internal states are presented in Table 2.

Both, self-injury during the past year before DBT treatment and lifetime self-injury were positively associated with ITP state dissociation (*r*_*s*_ = 0.35, 95% CI [0.03; 0.62], *BF*_*10*_ = 4.44; *r*_*s*_= 0.45, 95%CI[0.12; 0.66], *BF*_*10*_ = 3.59) and the Bayes factors indicated moderate evidence for these associations. State level of inner tension was only correlated with the number of lifetime self-injuries (*r*_*s*_ = 0.42, 95%CI[0.05; 0.71], *BF*_*10*_ = 1.5) and the evidence for this association can be considered anecdotal [46]. Affective Instability did not correlate with self-injury during the past year or lifetime self-injury whereas we found moderate evidence that the association with lifetime self-injury does not exist (*BF*_*10*_ = 0.21), the evidence for self-injuries during the past year is anecdotal (*BF*_*10*_ = 0.38) (see Table S8). The average number of days with suicidal ideation per week (i.e., frequency of suicidal ideation) during the past 6 month was not associated with any of the internal states (all *BF*_*10*_’s < 0.33) (See Table S8).

**Table 2:** Descriptive statistics

	M	SD	MDN
**Self-harm and borderline symptoms before admission**			
Suicidal ideation, frequency last 6 months ^a^	3.85	2.46	3.00
No. of lifetime self-injury	163.93	197.92	90.00
No. of self-injury last year	22.29	25.03	12.5
BSL-23	2.13	0.81	2.04
**Ecological Momentary Assessment Data (EMA)**			
Inner Tension	4.13	1.48	4.35
Affective Instability ^b^	2.81	1.58	2.61
State Dissociation	1.62	1.73	0.78

*Note. M* = mean, *SD* = standard deviation, *MDN* = median. *N* = 41. Ecological Momentary Assessment Data: Daily rating of inner tension and state dissociation on a Likert scale from 0 to 10 using a smartphone app. Affective instability: squared successive difference between consecutive ratings 1–2 h apart of inner tension. BSL-23: Borderline-Symptom List

^a^ Average number of days with suicidal ideation per week

^b^ One outlier with mean affective instability of 7.25 was identified and replaced with the trimmed mean = 2.7

### Self-injury, inner tension and state dissociation during DBT (cross-sectional analyses)

**Fig. 2:**
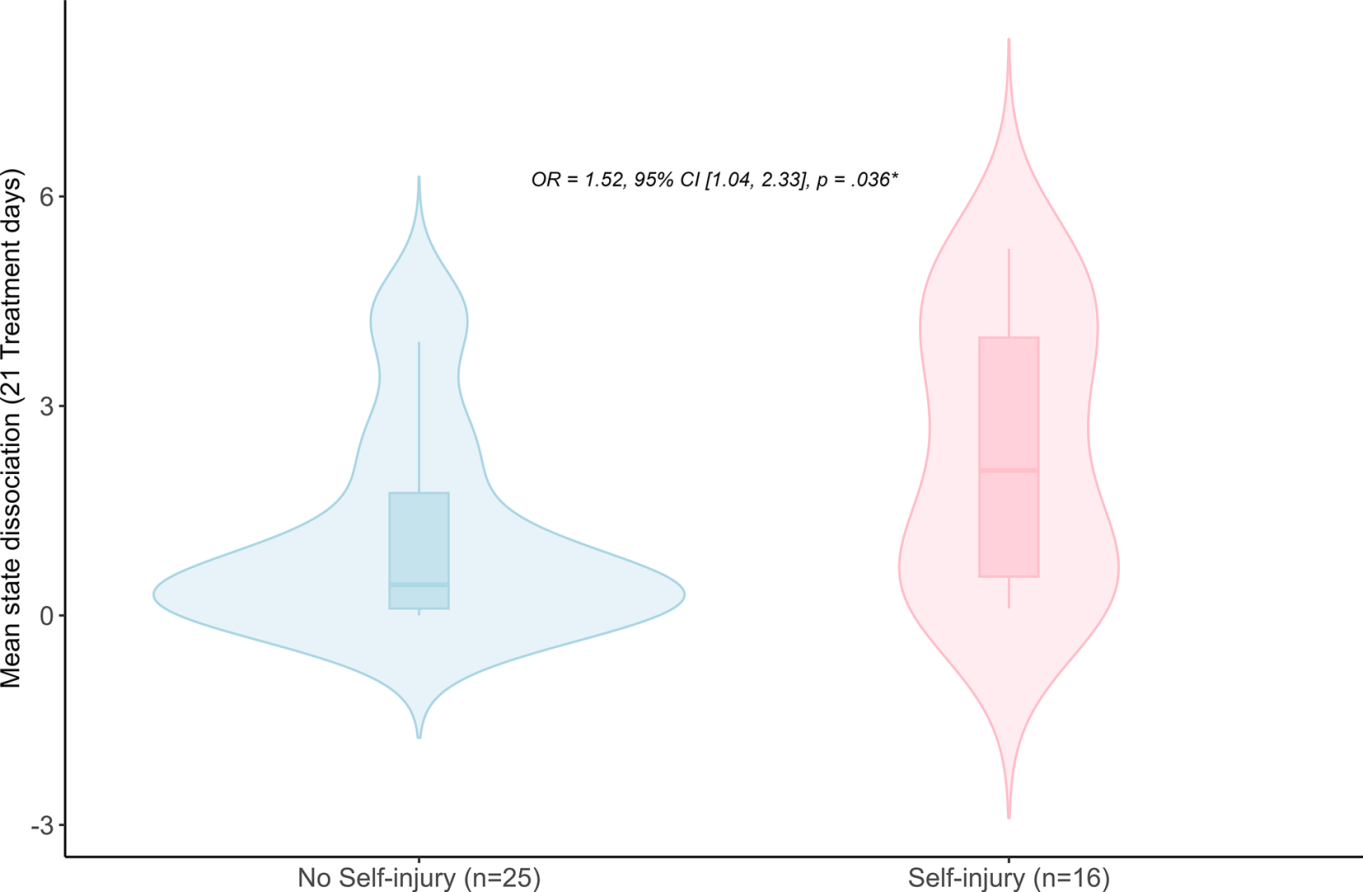
This figure illustrates violin plots of the participant-centered mean level of state dissociation assessed with EMA during the first three weeks of therapy depending on self-injury (during therapy, from patient’s files). It shows the distribution and boxplot for those who committed self-injury during an inpatient DBT treatment and those who did not commit self-injury during their therapy. Note that the smoothing kernel in R can cause slight negative oscillations in areas with sparse data. *OR*: odds ratio; CI: 95% confidence interval for standardized coefficients; *p* = *p* value; *n*: number of participants

39% (*N* = 16) of the sample engaged in self-injury during the inpatient DBT treatment, 6 patients had more than one self-injury (range: 2–4). Self-injury mostly occurred after the ITP/last day of compliant tracking at a median of 25 days after start of the tracking. Choosing self-injury primarily involved cutting with razor blades, knives, or sharp objects, and one patient severely harmed herself with her own nails. Higher ITP state dissociation increased the odds of self-injury during DBT, *OR* = 1.52, 95% CI [1.04,2.33], *p* = .036 with a small effect, suggesting that those with higher state dissociation are at an increased odds of engaging in self-injury compared to those with lower levels of state dissociation (see Fig. 2). The *BF*_*M*_ shows that the model including state dissociation is 1.87 times more likely than the null model (anecdotal evidence). Subjective ratings of inner tension (*OR* = 1.44, 95%CI [0.92,2.46], *p* = .138, *BF*_*M*_ = 0.77) and affective instability (*OR* = 1.05, 95% CI[0.66,1.66], *p* = .827, *BF*_*M*_ = 0.34) on the other hand showed no relationship to self-injurious behavior during DBT. For affective instability, the Bayes factor indicated that the null model was approximately three times more likely than the alternative model.

### Suicidal ideation, inner tension and state dissociation during DBT (longitudinal analyses)

**Fig. 3:**
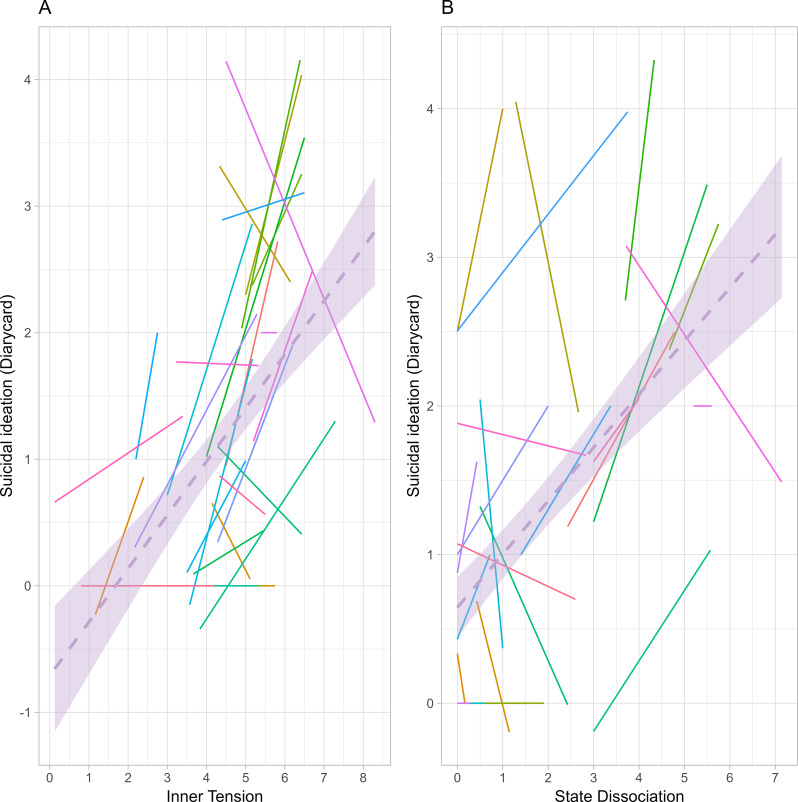
This figure illustrates overall regression lines (purple) and regression lines per participant for the daily association of internal states (assessed with a mobile app, aggregated per day) on the intensity of suicidal ideation (assessed in the diary card) across the first 21 monitored treatment days (excluding weekend days). The left figure (**A**) shows the association of daily levels of inner tension on suicidal ideation. While in most patients, days with higher inner tension were associated with higher suicidal ideation, some individuals also show a negative relationship (**A**). Figure (**B**) shows the influence of state dissociation on suicidal ideation. While there is overall a positive association, the graph indicates substantial variability between patients. Note that control variables of the LMMs are not considered in this figure

The average intensity of daily suicidal ideation ratings (Likert scale 0 to 5) was 1.08 (*SD* = 0.97, *MDN* = 0.89). A LMM revealed a significant and moderate to strong association of daily mean levels of inner tension and the intensity of suicidal ideation during DBT, β = 0.42 (95%CI: 0.20 to 0.64), *p* < .001 (see Table S9, Fig. 3A). Moreover, state dissociation was significantly related to the intensity of suicidal ideation, β = 0.38 (95%CI: 0.16 to 0.60), *p* = .002. However, this relationship revealed a high variability between patients (see Table S10, Fig. 3B). We found no association of affective instability and the intensity of suicidal ideation during DBT (see Table S11).

### Inner tension, state dissociation and symptom reduction during therapy

We found no effect of the ITP mean level of internal states on the reduction of borderline specific symptomology (BSL-23), considering baseline symptom severity (Table S12-S14). The model only including baseline symptom severity showed stronger evidence (*BF*_*M*_ = 2.08) than the model including baseline symptom severity and dissociation (*BF*_*M*_ = 0.98), suggesting that adding state dissociation does not improve the explained variance of symptom change during DBT. However, the Bayes factor (*BF*_*M*_ = 0.98) is close to one and therefore indicates no substantial evidence for or against the effect. For inner tension (*BF*_*M*_ = 2.32) and affective instability (*BF*_*M*_ = 2.10) the Bayes factors for the models only including symptom severity also received the strongest support.

## Discussion

We aimed to investigate fluctuations in internal psychological states during the first three weeks of an inpatient DBT program and how these are related to self-injury, suicidal ideation, and symptom reduction. This study contributes to the field by demonstrating that internal states begin to change already in the early stages of DBT, and that inner tension and state dissociation, which were closely monitored during inpatient DBT, are indeed associated with self-harming behaviors. We found that subjective state dissociation (mostly defined as derealization/depersonalization) and affective instability as well as average ratings of higher levels of inner tension (> 5, likely dysfunctional) decreased. Ratings in the lower inner tension levels (< 5) increased while overall inner tension remained unchanged during the initial three-weeks of DBT inpatient treatment. Across the day (9 a.m. to 9 p.m.), only state dissociation showed a slight increase from morning to midday, whereas inner tension and affective instability measures on average remained unchanged. Inner tension ratings were lower on weekends, while state dissociation and affective instability remained unaffected by weekend days. Self-injury in the year prior to DBT, lifetime self-injury, and self-injury occurring during inpatient DBT were associated with higher average dissociation levels during the first three-weeks of treatment. In contrast, average inner tension during DBT was only linked to lifetime self-injury. Days characterized by increased inner tension were closely associated with a higher intensity of suicidal ideation, whereas none of the internal states during therapy were associated with the frequency of suicidal ideation before therapy. Interestingly, affective instability showed no meaningful associations with self-injury or suicidal ideation before and during DBT. None of the average levels of state dissociation, inner tension, or affective instability predicted changes in borderline symptoms (BSL-23 T1-T0) over the course of DBT when baseline symptom severity (BSL T0) was controlled for.

Our results imply that state dissociation, affective instability, levels of inner tension are sensitive to changes in the initial therapy phase. This is consistent with previous research suggesting that dissociation tends to decrease during psychotherapy [47] and further indicates that improved emotion regulation may already begin to take effect early in therapy. However, the interpretation that patients increasingly apply emotion regulation skills learned during therapy, which may help reduce emotional arousal and prevent dissociative responses must remain tentative. In addition, therapeutic context factors, such as increased structure, support, and opportunities for guided regulation, may contribute to stabilization of emotional states over time. Future research should examine these mechanisms more directly, for example by linking EMA assessments of dissociation and inner tension to momentary skill use or experimental measures of emotion regulation. Our findings further support the notion that the reduction of high, potentially mainly dysfunctional levels of inner tension rather than the overall levels could be a marker of therapeutic progress. Moreover, during psychotherapy — especially in the context of trauma exposure — a specific, moderate level of inner tension is considered desirable [48], which may also be reflected in our findings of lower inner tension levels on weekends compared to weekdays when therapeutic sessions took place. The increase in low inner tension levels may also be related to the requirements of the treatment setting, which actively promotes engagement and activity in line with previous studies showing that the level of physical activity among psychiatric patients increases during inpatient treatment [49].

As we observed an increase in dissociative symptoms from morning to midday, timing interventions to anticipate midday increases in dissociation—such as grounding techniques—may help patients remain engaged and reduce the risk of dissociation interfering with therapy. Given that research has shown a strong association between sleep and dissociative symptoms [50, 51], the increase in dissociation observed around midday could potentially be related to rising levels of sleepiness during that time. Alternatively, it may also reflect the accumulation of emotional stressors or interpersonal demands that typically intensify as the day progresses. Although we had only limited descriptive data for the later evening hours and patients were not explicitly asked to track these times in our study, it is interesting that the relatively few ratings of mean inner tension and state dissociation were particularly high at nighttime. One long-term study using wrist actigraphy over 28 days found that BPD patients were significantly more active in the late evening compared to healthy controls points to a delayed circadian rhythm as a potentially meaningful clinical feature in BPD [52]. Future studies could therefore extend the monitoring window to include evening and nighttime hours, systematically assess triggers for inner tension and state dissociation to identify potential influencing factors, and ideally incorporate physiological parameters to allow for comparisons between subjective and more objective data.

Our findings that increased life-time, pre-therapy, and in-therapy self-injury relates to higher in-therapy state dissociation aligns with the literature showing that patients with dissociative disorders are more likely to report non-suicidal self-injury (NSSI) compared to non-dissociative psychiatric patients [53]. It also aligns with the finding, that adults recruited from the local community who engaged in NSSI experienced higher levels of dissociation compared to healthy adult controls [54]. In a clinical sample of 91 outpatient women with BPD and comorbid eating disorders, higher levels of dissociation were significantly associated with a greater number of NSSI episodes in the past six months and this effect depended on the level of cognitive reappraisal, particularly weakening the association between dissociation and NSSI [55]. While these studies have shown associations between self-reported past self-injury and dissociation, we were able to demonstrate that, in a sample of individuals with BPD undergoing inpatient DBT, self-reported state dissociation levels over a three-week period were not only related to self-reported lifetime and pre-therapy self-injury but also to clinician-assessed self-injury occurring during therapy. This supports theories suggesting that one function of self-injury could be to regain a sense of control and reconnect with the self when dissociation occurs [15]. While these findings suggest that state dissociation relates to more dysfunctional behavior, we did not observe a negative impact of state dissociation on the reduction of overall borderline symptoms during standard DBT—unlike prior findings showing a detrimental effect of dissociation on PTSD symptom change in DBT-PTSD [29, 30]. This may indicate that when dissociation is explicitly and intensely addressed within standard DBT, for example through behavioral or chain analyses, as it was the case in our sample, it does not necessarily impair therapeutic success regarding overall BPD symptoms.

Our observation, that individuals with higher levels of state dissociation submitted a higher number of ratings particularly in the second half of the observation period, indicates that patients with problematic dissociative symptoms followed therapeutic instructions to actively decrease these symptoms which included a more intense self-monitoring compared to patients with no or low dissociative symptoms. Another naturalistic study also found that dissociation scores have little influence on the prediction of symptom change but that the change in dissociation is significantly associated with change in scores of PTSD, depression, and suicidality [47]. This can be taken as another indicator that the effective treatment of dissociation might influence various other symptoms. However, although our Bayesian analysis favored a model including only baseline borderline symptom severity, the model that included both baseline severity and state dissociation yielded a Bayes factor close to 1, indicating that a potentially negative influence of dissociation on therapeutic success cannot be ruled out. Due to our small sample size and the naturalistic study design, these findings require replication in larger, controlled studies such as the exploration of how changes in dissociation influence other symptom domains. Moreover, we only found small to moderate effect sizes for the association of self-injury and dissociation indicating that self-injury might only be one of several possible mechanisms to handle dissociation or vice versa. Further, as Navarro-Haro et al., [55] suggested, the effect of dissociation on self-injury could also depend on individual emotion regulation strategies.

Interestingly, we only found an association between inner tension and lifetime self-injury, but not with pre-therapy and in-therapy self-injury. Clinical observation and previous studies have shown that self-injury effectively reduces both subjectively and objectively experienced stress [3]. While our data did not allow us to clarify temporal dynamics to better understand the direct and long-term effect of self-injury on inner tension, one hypothesis could be that individuals who have frequently engaged in self-injury report lower current inner tension levels because their subjective distress is effectively alleviated through these behaviors. However, the low base rate of self-injury during inpatient treatment limits the reliability of our data in this respect. Finally, self-injury is assumed to serve multiple functions [14], and state dissociation and inner tension may not only represent symptoms interacting with self-injury but might also act as independent coping strategies in the context of dysfunctional emotion regulation.

Our longitudinal analyses further showed that days with higher intensity of suicidal ideation were also characterized by increased state dissociation and inner tension. During dissociative states or periods of high inner tension, perception and thinking are often narrowed or fragmented, which may severely impair problem-solving abilities and cognitive flexibility. As a result, suicidal ideation could emerge as the only perceived solution and a way to provide relief, according to the “escape theory” suggesting self-harm and suicidal ideation as attempts to escape from aversive self-awareness and intolerable emotional states when problem-solving is lacking [56]. Consistent with this, De Beurs et al., [25] found that internal entrapment, the feeling of being trapped by one’s own thoughts and emotions, shows a very strong association with suicidal ideation. High levels of dissociation may trigger an immediate urge to act in order to regain a sense of connection, thereby being more strongly associated with self-injury. Likely, there is a dynamical interaction between dissociation, intense inner tension and self-harm and inner tension or state dissociation could also be a response to self-harming behavior.

Finally, we found no association of affective instability (i.e., squared successive difference between consecutive ratings of inner tension) with lifetime, one year pre-therapy and in-therapy self-injury or suicidal ideation. Concordantly, Links et al., [12] found that negative mood intensity, but not affective instability was significantly related to the intensity of self-reported suicide ideation and the past number of suicide attempts. Contrary, another study found that variability in suicidal ideation is linked to affective instability [57]. It may be important to consider whether predictive effects differ depending on whether overall daily means or individual momentary peak values are analyzed. Given our approach, it is conceivable that single high-intensity moments could be predictive, even if the overall daily mean remains non-significant.

### Limitations

A limitation of the study is that state dissociation was assessed using a single EMA item. This approach does not capture the multidimensional nature of dissociation and precludes the estimation of reliability. Although patients were provided with a definition and examples, it remains unclear which aspects of state dissociation were referenced when responding. Moreover, the present EMA design did not allow reliable testing of time-lagged relationships between dissociation, inner tension, and self-injurious behavior due to limited temporal resolution and low self-injury frequency. A further limitation is that the observed reductions in inner tension and state dissociation cannot be attributed specifically to DBT. Changes over time may also reflect the structured inpatient environment, non-specific therapeutic effects, or natural symptom fluctuations during the course of treatment. Future research would benefit from larger samples, more systematic tracking of external events, more objective assessments of dissociation, the inclusion of a control group, and, crucially, higher-frequency assessments to better capture the temporal dynamics between internal states like inner tension and dissociation and self-harming behaviors.

## Conclusion

Our study demonstrates that state dissociation, high levels of inner tension and affective instability can decrease already within the first three weeks of DBT. Identifying and anticipating increases of state dissociation towards midday may help to prevent its disruptive impact on therapy. Individuals who report higher levels of state dissociation during the early phase of DBT are more likely to injure themselves—lifetime, one year pre-therapy, and during therapy—though our findings do not support the hypothesis that state dissociation impairs borderline symptom improvement during standard DBT. While average inner tension during early therapy is not associated with in-therapy self-injury, it is linked to lifetime self-injury. On a daily level, higher ratings of inner tension, as well as state dissociation, indicate increased intensity of suicidal ideation during treatment or vice versa, emphasizing the utility of monitoring state dissociation and inner tension in self-harm prevention.

## Supplementary Information

Below is the link to the electronic supplementary material.

Supplementary Material 1

## Data Availability

Preregistration: [https://osf.io/dfq9y/overview?view%5C_only=4c19b891bb6448009b22f60b2552bd73]. Further data from the reported study is available (https://cloudstore.uni-ulm.de/s/BH3Xotxb8TLKKm9) and supplementary material detailing the statistical analyses is provided.
