# Supplementary material for: Fluctuations of dissociation and inner tension during inpatient dialectical behavior therapy: associations with self-injury and suicidal ideation
Source: Borderline Personal Disord Emot Dysregul. 2026 Mar 6;13:8. doi: 10.1186/s40479-026-00339-1 (PMC13064321; doi:10.1186/s40479-026-00339-1)

**Fluctuations of Dissociation and Inner Tension during Inpatient Dialectical Behavior Therapy: Associations with Self-Injury and Suicidal Ideation**

| **Table S1**  *Diagnoses of the inpatient sample (n=41)* | | |
| --- | --- | --- |
|  | *n* | *%* |
| Main Diagnoses |  |  |
| Emotional unstable PD, borderline type | 37 | 90.24 |
| Emotional unstable PD, impulsive type | 2 | 04.88 |
| Combined PD with borderline traits | 2 | 04.88 |
| Comorbid diagnoses |  |  |
| Depression | 26 | 63.41 |
| Posttraumatic Stress Disorders | 17 | 41.46 |
| Substance Use Disorders | 4 | 09.76 |
| Hyperkinetic Disorders | 4 | 09.76 |
| Other anxiety Disorders | 3 | 07.32 |
| Eating Disorders | 2 | 04.88 |
| *Note.*  PD: Personality disorder. Psychiatric diagnosis according to the International Statistical Classification of Diseases and Related Health Problems (ICD-10). Participants had on average one further comorbid diagnoses (*M* = 1.51; *SD* = 1.05, *MDN* = 1). Other anxiety disorders include Chronic pain disorder with somatic and psychological factors (*n* = 2) and social phobia (*n* = 1). | | |

| **Table S2** | **Inner tension** | | | | | |
| --- | --- | --- | --- | --- | --- | --- |
| *Predictors* | *Estimates* | *std. Beta* | *CI* | *standardized CI* | *p* | *df* |
| (Intercept) | 4.05 | -0.11 | 3.58 – 4.53 | -0.34 – 0.12 | **<0.001** | 39.43 |
| N | 0.12 | 0.04 | -0.02 – 0.27 | -0.01 – 0.08 | 0.088 | 3118.51 |
| measurement day | -0.00 | -0.00 | -0.06 – 0.06 | -0.03 – 0.03 | 0.922 | 3114.84 |
| weekend [1] | -0.16 | -0.08 | -0.28 – -0.03 | -0.14 – -0.02 | **0.014** | 3092.09 |
| **Random Effects** | | | | | | |
| σ^2^ | 2.23 | | | | | |
| τ_00_ _Participant_ID_ | 1.98 | | | | | |
| ICC | 0.47 | | | | | |
| N _Participant_ID_ | 41 | | | | | |
| Observations | 3128 | | | | | |
| Marginal R^2^ / Conditional R^2^ | 0.003 / 0.472 | | | | | |

**Table S2-S4**: Inner tension, state dissociation, and affective instability throughout the initial three weeks of DBT. N = total number of ratings. Measurement day = DBT treatment day (continuous variable). Weekend (1=weekend, 0=no weekend). Continuous variables are z-transformed (mean = 0, sd =1).

| **Table S3** | **State dissociation** | | | | | | |
| --- | --- | --- | --- | --- | --- | --- | --- |
| *Predictors* | *Estimates* | *std. Beta* | *CI* | *standardized CI* | *p* | *std. p* | *df* |
| (Intercept) | 1.40 | 0.02 | 0.84 – 1.97 | -0.24 – 0.28 | **<0.001** | 0.876 | 40.18 |
| N | 0.23 | 0.05 | 0.10 – 0.36 | 0.02 – 0.09 | **<0.001** | **0.002** | 3114.33 |
| measurement day | -0.25 | -0.03 | -0.35 – -0.14 | -0.05 – -0.01 | **<0.001** | **0.004** | 3096.60 |
| weekend [1] | -0.01 | -0.00 | -0.10 – 0.09 | -0.05 – 0.04 | 0.894 | 0.894 | 3086.62 |
| N × measurement day | 0.16 | 0.04 | 0.07 – 0.25 | 0.02 – 0.07 | **0.001** | **0.001** | 3094.46 |
| **Random Effects** | | | | | | | |
| σ^2^ | 1.29 | | | | | | |
| τ_00_ _Participant_ID_ | 3.05 | | | | | | |
| ICC | 0.70 | | | | | | |
| N _Participant_ID_ | 41 | | | | | | |
| Observations | 3128 | | | | | | |
| Marginal R^2^ / Conditional R^2^ | 0.005 / 0.704 | | | | | | |

| **Table S4** | **Affective instability** | | | | | |
| --- | --- | --- | --- | --- | --- | --- |
| *Predictors* | *Estimates* | *std. Beta* | *CI* | *standardized CI* | *p* | *df* |
| (Intercept) | 2.66 | 0.02 | 1.95 – 3.37 | -0.08 – 0.11 | **<0.001** | 44.64 |
| N | -0.05 | -0.01 | -0.54 – 0.43 | -0.07 – 0.05 | 0.826 | 661.25 |
| measurement day | -0.47 | -0.09 | -0.70 – -0.25 | -0.13 – -0.05 | **<0.001** | 2136.24 |
| weekend [1] | -0.13 | -0.03 | -0.60 – 0.34 | -0.12 – 0.07 | 0.582 | 2369.00 |
| **Random Effects** | | | | | | |
| σ^2^ | 24.45 | | | | | |
| τ_00_ _Participant_ID_ | 1.54 | | | | | |
| ICC | 0.06 | | | | | |
| N _Participant_ID_ | 41 | | | | | |
| Observations | 2373 | | | | | |
| Marginal R^2^ / Conditional R^2^ | 0.008 / 0.067 | | | | | |

**Table S5-S7**. Inner tension, state dissociation, and affective instability across different day times for hours (factor; categorical variable) with reminders to rate the momentary level

| **Table S5 Inner tension** | | | | | |  |
| --- | --- | --- | --- | --- | --- | --- |
| *Predictors* | *Estimates* | *std. Beta* | *CI* | *standardized CI* | *p* | *df* |
| (Intercept) | 4.01 | -0.18 | 3.52 – 4.49 | -0.43 – 0.07 | **<0.001** | 45.98 |
| hour [11] | 0.11 | 0.05 | -0.11 – 0.32 | -0.06 – 0.16 | 0.338 | 2431.40 |
| hour [13] | 0.17 | 0.09 | -0.05 – 0.40 | -0.03 – 0.20 | 0.127 | 2431.58 |
| hour [15] | 0.15 | 0.07 | -0.07 – 0.36 | -0.04 – 0.19 | 0.190 | 2432.72 |
| hour [17] | 0.09 | 0.05 | -0.12 – 0.30 | -0.06 – 0.15 | 0.403 | 2431.40 |
| hour [19] | 0.06 | 0.03 | -0.16 – 0.28 | -0.08 – 0.14 | 0.581 | 2432.21 |
| hour [21] | -0.09 | -0.05 | -0.31 – 0.13 | -0.16 – 0.07 | 0.430 | 2432.54 |
| measurement day | -0.00 | -0.00 | -0.07 – 0.06 | -0.03 – 0.03 | 0.901 | 2455.49 |
| **Random Effects** | | | | | | |
| σ^2^ | 2.12 | | | | | |
| τ_00_ _Participant_ID_ | 2.06 | | | | | |
| ICC | 0.49 | | | | | |
| N _Participant_ID_ | 41 | | | | | |
| Observations | 2479 | | | | | |
| Marginal R^2^ / Conditional R^2^ | 0.002 / 0.494 | | | | | |

| **Table S6** | **state dissociation** | | | | | |
| --- | --- | --- | --- | --- | --- | --- |
| *Predictors* | *Estimates* | *std. Beta* | *CI* | *standardized CI* | *p* | *df* |
| (Intercept) | 1.51 | -0.01 | 0.93 – 2.08 | -0.29 – 0.26 | **<0.001** | 42.65 |
| hour [11] | 0.18 | 0.08 | 0.02 – 0.34 | 0.01 – 0.16 | **0.032** | 2431.66 |
| hour [13] | 0.27 | 0.13 | 0.10 – 0.44 | 0.05 – 0.21 | **0.002** | 2431.70 |
| hour [15] | 0.15 | 0.07 | -0.02 – 0.31 | -0.01 – 0.15 | 0.080 | 2432.26 |
| hour [17] | 0.14 | 0.06 | -0.02 – 0.29 | -0.01 – 0.14 | 0.092 | 2431.53 |
| hour [19] | 0.13 | 0.06 | -0.03 – 0.30 | -0.02 – 0.14 | 0.114 | 2431.99 |
| hour [21] | 0.11 | 0.05 | -0.06 – 0.28 | -0.03 – 0.13 | 0.192 | 2432.14 |
| measurement day | -0.08 | -0.04 | -0.13 – -0.03 | -0.06 – -0.02 | **0.001** | 2442.58 |
| **Random Effects** | | | | | | |
| σ^2^ | 1.20 | | | | | |
| τ_00_ _Participant_ID_ | 3.18 | | | | | |
| ICC | 0.73 | | | | | |
| N _Participant_ID_ | 41 | | | | | |
| Observations | 2479 | | | | | |
| Marginal R^2^ / Conditional R^2^ | 0.003 / 0.726 | | | | | |

| **Table S7** | **Affective Instability** | | | | | |
| --- | --- | --- | --- | --- | --- | --- |
| *Predictors* | *Estimates* | *std. Beta* | *CI* | *standardized CI* | *p* | *df* |
| (Intercept) | 2.06 | -0.09 | 1.12 – 2.99 | -0.28 – 0.11 | **<0.001** | 647.16 |
| hour [11] | 0.67 | 0.14 | -0.33 – 1.67 | -0.07 – 0.35 | 0.187 | 2056.42 |
| hour [13] | 0.32 | 0.07 | -0.69 – 1.33 | -0.14 – 0.28 | 0.531 | 2055.82 |
| hour [15] | 0.54 | 0.11 | -0.47 – 1.54 | -0.10 – 0.32 | 0.294 | 2056.03 |
| hour [17] | 0.37 | 0.08 | -0.61 – 1.35 | -0.13 – 0.28 | 0.457 | 2057.57 |
| hour [19] | 0.42 | 0.09 | -0.57 – 1.41 | -0.12 – 0.29 | 0.403 | 2058.66 |
| hour [21] | 0.63 | 0.13 | -0.37 – 1.64 | -0.08 – 0.34 | 0.216 | 2059.56 |
| measurement day | -0.36 | -0.07 | -0.58 – -0.14 | -0.12 – -0.03 | **0.001** | 1959.98 |
| **Random Effects** | | | | | | |
| σ^2^ | 21.85 | | | | | |
| τ_00_ _Participant_ID_ | 1.31 | | | | | |
| ICC | 0.06 | | | | | |
| N _Participant_ID_ | 41 | | | | | |
| Observations | 2070 | | | | | |
| Marginal R^2^ / Conditional R^2^ | 0.007 / 0.063 | | | | | |

**Table S8**. Correlation Self-harm and internal states before DBT

| *Spearman's Correlations* | | | | | | | | | | | | | | | | | | | | | |
| --- | --- | --- | --- | --- | --- | --- | --- | --- | --- | --- | --- | --- | --- | --- | --- | --- | --- | --- | --- | --- | --- |
| Variable | | | | |  | | | Self-injury last year | | | | State Dissociation | | Lifetime Self-injury | | Inner Tension | | Affective Instability | | Suicidal thoughts | |
|  |  |  | |  | |  |  | |  | |  |  |  |  |  |  |  |  |  |  |  |
|  |  |  | |  | |  |  | |  | |  |  |  |  |  |  |  |  |  |  |  |
| 2. State Dissociation | | |  | | Spearman's rho | |  | 0.354 | |  | | — |  |  |  |  |  |  |  |  |  |
|  | | |  | | p-value | |  | 0.031 | |  | | — |  |  |  |  |  |  |  |  |  |
|  | | |  | | Lower 95% CI | |  | 0.058 | |  | | — |  |  |  |  |  |  |  |  |  |
|  | | |  | | Upper 95% CI | |  | 0.631 | |  | | — |  |  |  |  |  |  |  |  |  |
| 3. Lifetime Self-injury | | |  | | Spearman's rho | |  | 0.572 | |  | | 0.448 |  | — |  |  |  |  |  |  |  |
|  | | |  | | p-value | |  | < .001 | |  | | 0.007 |  | — |  |  |  |  |  |  |  |
|  | | |  | | Lower 95% CI | |  | 0.330 | |  | | 0.148 |  | — |  |  |  |  |  |  |  |
|  | | |  | | Upper 95% CI | |  | 0.738 | |  | | 0.669 |  | — |  |  |  |  |  |  |  |
| 4. Inner Tension | | |  | | Spearman's rho | |  | 0.209 | |  | | 0.565 |  | 0.415 |  | — |  |  |  |  |  |
|  | | |  | | p-value | |  | 0.214 | |  | | < .001 |  | 0.013 |  | — |  |  |  |  |  |
|  | | |  | | Lower 95% CI | |  | -0.128 | |  | | 0.313 |  | 0.053 |  | — |  |  |  |  |  |
|  | | |  | | Upper 95% CI | |  | 0.498 | |  | | 0.744 |  | 0.689 |  | — |  |  |  |  |  |
| 5. Affective Instability | | |  | | Spearman's rho | |  | -0.159 | |  | | 0.005 |  | -0.045 |  | -0.103 |  | — |  |  |  |
|  | | |  | | p-value | |  | 0.346 | |  | | 0.977 |  | 0.795 |  | 0.523 |  | — |  |  |  |
|  | | |  | | Lower 95% CI | |  | -0.527 | |  | | -0.288 |  | -0.417 |  | -0.425 |  | — |  |  |  |
|  | | |  | | Upper 95% CI | |  | 0.207 | |  | | 0.320 |  | 0.304 |  | 0.204 |  | — |  |  |  |
| 6. Suicidal thoughts | | |  | | Spearman's rho | |  | 0.208 | |  | | 0.036 |  | 0.045 |  | 0.105 |  | -0.139 |  | — |  |
|  | | |  | | p-value | |  | 0.245 | |  | | 0.839 |  | 0.810 |  | 0.554 |  | 0.434 |  | — |  |
|  | | |  | | Lower 95% CI | |  | -0.144 | |  | | -0.337 |  | -0.264 |  | -0.248 |  | -0.478 |  | — |  |
|  | | |  | | Upper 95% CI | |  | 0.544 | |  | | 0.432 |  | 0.371 |  | 0.459 |  | 0.225 |  | — |  |
|  | | | | | | | | | | | | | | | | | | | | | |
| *Note.*  Confidence intervals based on 1000 bootstrap replicates. | | | | | | | | | | | | | | | | | | | | | |

**Table S9-S11.** Daily levels of Inner tension, state dissociation, and affective instability and its association with daily levels of suicidal ideation (diarycard ratings)

| **Table S9. Diarycard suicidal ideation and inner tension** | | | | | |  |
| --- | --- | --- | --- | --- | --- | --- |
| *Predictors* | *Estimates* | *std. Beta* | *CI* | *standardized CI* | *p* | *df* |
| (Intercept) | 1.12 | -0.03 | 0.78 – 1.46 | -0.30 – 0.23 | **<0.001** | 30.17 |
| Inner tension mean | 0.56 | 0.42 | 0.26 – 0.85 | 0.20 – 0.64 | **0.001** | 19.81 |
| measurement day | -0.01 | -0.01 | -0.12 – 0.11 | -0.10 – 0.08 | 0.891 | 161.16 |
| **Random Effects** | | | | | | |
| σ^2^ | 0.51 | | | | | |
| τ_00_ _Participant_ID_ | 0.72 | | | | | |
| τ_11_ _Participant_ID.scale(anspann1_mean)_ | 0.27 | | | | | |
| ρ_01_ _Participant_ID_ | 0.29 | | | | | |
| ICC | 0.66 | | | | | |
| N _Participant_ID_ | 35 | | | | | |
| Observations | 193 | | | | | |
| Marginal R^2^ / Conditional R^2^ | 0.164 / 0.717 | | | | | |

| **Table S10. Diarycard suicidal ideation and state dissociation** | | | | | |  |
| --- | --- | --- | --- | --- | --- | --- |
| *Predictors* | *Estimates* | *std. Beta* | *CI* | *standardized CI* | *p* | *df* |
| (Intercept) | 1.18 | -0.02 | 0.84 – 1.51 | -0.27 – 0.24 | **<0.001** | 28.53 |
| State dissociation mean | 0.48 | 0.38 | 0.20 – 0.76 | 0.16 – 0.60 | **0.002** | 20.77 |
| measurement day | 0.03 | 0.02 | -0.10 – 0.15 | -0.08 – 0.12 | 0.689 | 168.50 |
| **Random Effects** | | | | | | |
| σ^2^ | 0.62 | | | | | |
| τ_00_ _Participant_ID_ | 0.73 | | | | | |
| τ_11_ _Participant_ID.scale(diss1_mean)_ | 0.06 | | | | | |
| ρ_01_ _Participant_ID_ | -0.02 | | | | | |
| ICC | 0.56 | | | | | |
| N _Participant_ID_ | 35 | | | | | |
| Observations | 193 | | | | | |
| Marginal R^2^ / Conditional R^2^ | 0.145 / 0.624 | | | | | |

| **Table S11. Diarycard suicidal ideation and affective instability (SSD)** | | | | | | |
| --- | --- | --- | --- | --- | --- | --- |
| *Predictors* | *Estimates* | *std. Beta* | *CI* | *standardized CI* | *p* | *df* |
| (Intercept) | 1.22 | -0.01 | 0.83 – 1.61 | -0.31 – 0.29 | **<0.001** | 34.08 |
| SSD mean | 0.06 | 0.04 | -0.11 – 0.23 | -0.07 – 0.15 | 0.480 | 110.15 |
| measurement day | -0.00 | -0.00 | -0.13 – 0.12 | -0.10 – 0.10 | 0.967 | 161.50 |
| **Random Effects** | | | | | | |
| σ^2^ | 0.63 | | | | | |
| τ_00_ _Participant_ID_ | 1.10 | | | | | |
| τ_11_ _Participant_ID.scale(tense_arousal_SSD_mean)_ | 0.00 | | | | | |
| ρ_01_ _Participant_ID_ | 1.00 | | | | | |
| ICC | 0.64 | | | | | |
| N _Participant_ID_ | 35 | | | | | |
| Observations | 191 | | | | | |
| Marginal R^2^ / Conditional R^2^ | 0.002 / 0.636 | | | | | |

**Table S12-S14.** The influence of mean ITP Inner tension, state dissociation, and affective instability on the change in borderline-specific symptoms (Δ BSL-23), accounting for baseline symptom severity (BSL-23 at admission) from admission to discharge

| **Table S12** | Δ BSL-23 | | | | | |
| --- | --- | --- | --- | --- | --- | --- |
| *Predictors* | *Estimates* | *std. Beta* | *CI* | *standardized CI* | *p* | *df* |
| (Intercept) | 30.31 | 0.00 | 24.20 – 36.42 | -0.34 – 0.34 | **<0.001** | 30.00 |
| BSL Mittel T1 | 8.26 | 0.46 | 0.97 – 15.55 | 0.05 – 0.86 | **0.028** | 30.00 |
| State dissociation | -3.25 | -0.19 | -10.31 – 3.80 | -0.59 – 0.22 | 0.354 | 30.00 |
| Observations | 33 | | | | | |
| R^2^ / R^2^ adjusted | 0.154 / 0.097 | | | | | |
|  |  | | | | | |
| **Table S13** | Δ BSL-23 | | | | | |
| *Predictors* | *Estimates* | *std. Beta* | *CI* | *standardized CI* | *p* | *df* |
| (Intercept) | 30.41 | -0.00 | 24.22 – 36.61 | -0.34 – 0.34 | **<0.001** | 30.00 |
| BSL Mittel T1 | 7.15 | 0.40 | -0.17 – 14.47 | -0.01 – 0.80 | 0.055 | 30.00 |
| Inner tension | -1.39 | -0.07 | -9.29 – 6.51 | -0.48 – 0.33 | 0.722 | 30.00 |
| Observations | 33 | | | | | |
| R^2^ / R^2^ adjusted | 0.133 / 0.075 | | | | | |

| **Table S14** | Δ BSL-23 | | | | | |
| --- | --- | --- | --- | --- | --- | --- |
| *Predictors* | *Estimates* | *std. Beta* | *CI* | *standardized CI* | *p* | *df* |
| (Intercept) | 30.49 | -0.00 | 24.37 – 36.62 | -0.34 – 0.34 | **<0.001** | 30.00 |
| BSL Mittel T1 | 6.77 | 0.37 | 0.52 – 13.01 | 0.03 – 0.72 | **0.035** | 30.00 |
| Affective instability | 3.02 | 0.15 | -3.96 – 10.00 | -0.20 – 0.49 | 0.384 | 30.00 |
| Observations | 33 | | | | | |
| R^2^ / R^2^ adjusted | 0.151 / 0.094 | | | | | |

Figure S1. Heatplot of inner tension. The x-axis represents individual participants, while the y-axis displays the measurement days, separated by dashed lines, and the specific hours at which reminders were scheduled (from 9 a.m. to 9 p.m.; i.e., 9, 11, 13, 15, 17, 19, and 21 o'clock).Each rectangle corresponds to a rating provided by a specific participant on a specific measurement day at a specific time. White cells indicate missing data, whereas lighter colors represent lower values and darker colors indicate more intense or pronounced values.


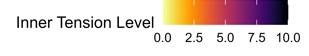


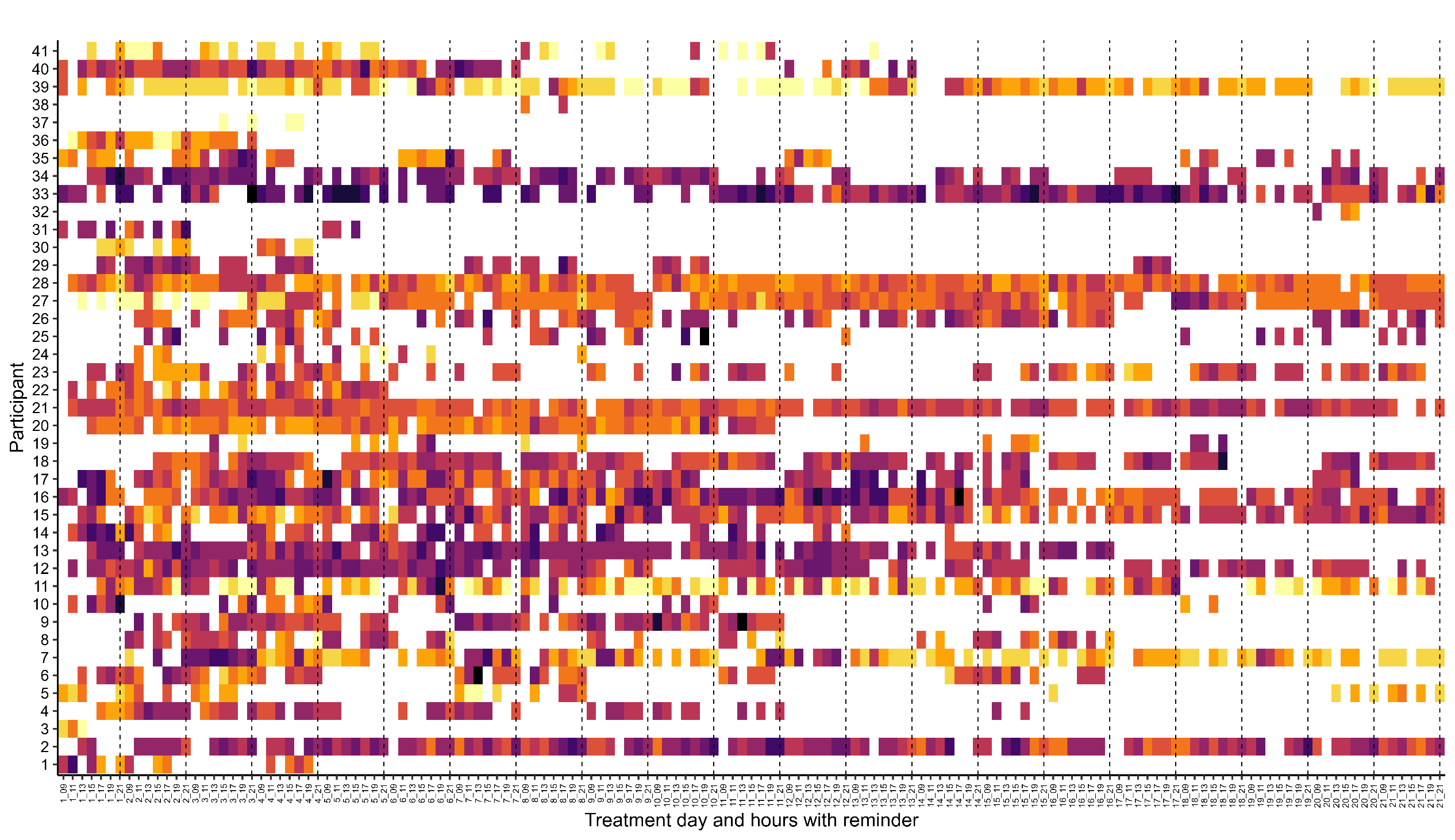


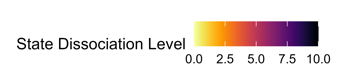
Figure S2. Heatplot of state dissociation. The x-axis represents individual participants, while the y-axis displays the measurement days, separated by dashed lines, and the specific hours at which reminders were scheduled (from 9 a.m. to 9 p.m.; i.e., 9, 11, 13, 15, 17, 19, and 21 o'clock).Each rectangle corresponds to a rating provided by a specific participant on a specific measurement day at a specific time. White cells indicate missing data, whereas lighter colors represent lower values and darker colors indicate more intense or pronounced values.


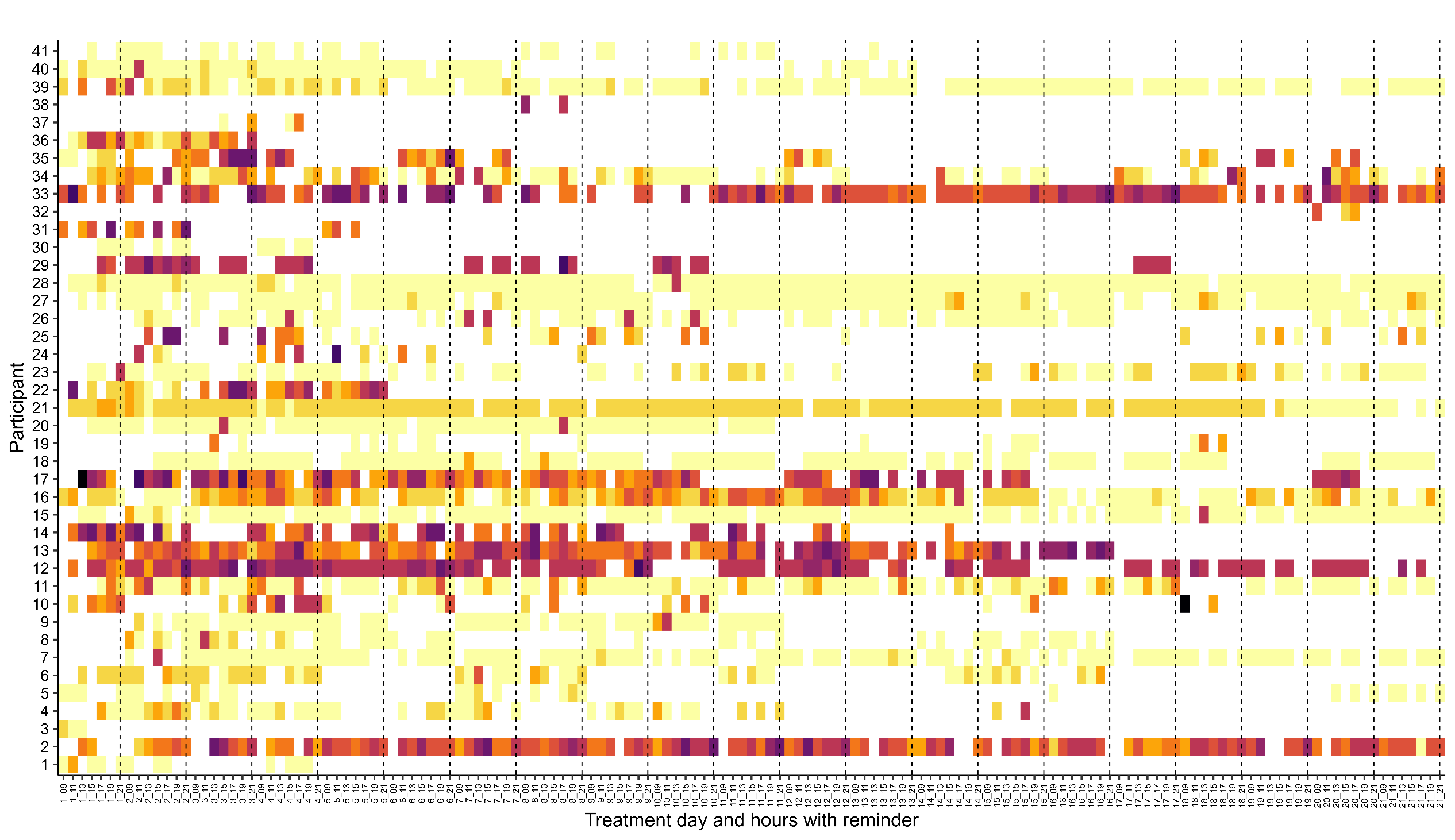

Supplement: Supplementary file 1 — Supplementary Material 1 [file 40479_2026_339_MOESM1_ESM.docx]
